# Spatiotemporal distribution and determinants of open defecation among households in Ethiopia: A *Mixed effect and spatial analysis*

**DOI:** 10.1371/journal.pone.0268342

**Published:** 2022-05-19

**Authors:** Daniel Gashaneh Belay, Dagmawi Chilot, Melaku Hunie Asratie

**Affiliations:** 1 Department of Human Anatomy, School of Medicine, College of Medicine and Health Sciences, University of Gondar, Gondar, Ethiopia; 2 Department of Epidemiology and Biostatistics, Institute of Public Health, College of Medicine and Health Sciences, University of Gondar, Gondar, Ethiopia; 3 Addis Ababa University, College of Health Sciences, Center for Innovative Drug Development and Therapeutic Trials for Africa (CDT-Africa), Addis Ababa, Ethiopia; 4 Department of Human Physiology, University of Gondar, College of Medicine and Health Science, School of Medicine, Gondar, Ethiopia; 5 Department of Women’s and Family Health, School of Midwifery, College of Medicine and Health Sciences, University of Gondar, Gondar, Ethiopia; Universidade Lisboa, Instituto superior Técnico, PORTUGAL

## Abstract

**Background:**

Open defecation is the disposal of human faeces in the fields, forests, bushes, and open bodies of water. It is practiced more in sub-Saharan African countries and is considered a sign of underdevelopment. Open defecation facilitates the transmission of pathogens that cause diarrheal diseases which is the second leading contributor to the global burden of disease. In Ethiopia, it kills half a million under-five children annually. Even though open defecation practice is a major cause of childhood mortality and morbidity in Ethiopia, there is minimal evidence on the trend, spatiotemporal distribution, wealth-related inequalities, and other determinates of open defecation practice.

**Objectives:**

Therefore, this study aimed to investigate the trend, spatiotemporal distribution, and determinants of open defecation among households in Ethiopia.

**Methods:**

Cross-sectionally collected secondary data analysis was conducted based on 2016 Ethiopian Demographic and Health Survey (EDHS). A total weighted sample of 16,554 households was included. We assessed the 16 years (2000–2016) trend of open defecation with 95% confidence intervals. Data were weighted, recoded, cleaned, and analyzed using STATA version 14.2 software. A mixed-effect analysis was employed to identify factors contributing to open defecation practice in Ethiopia. In the final multivariable analysis, the associations between dependent and independent variables were presented using adjusted odds ratios and 95% confidence intervals with a p-value of <0.05. The concentration index was used to assess wealth-related inequalities, while spatial analysis was used to explore the spatial distribution and significant windows of open defecation practice.

**Results:**

The trend of open defecation practice in Ethiopia was significantly decreased from 81.96% (95% CI: 81.08, 82.8) in 2000 EDHS, to 32.23% (95% CI: 31.16, 33.31) in 2016 EDHS. Individual-level factors such as; age, educational attainment, marital status, media exposure, wealth status, and source of drinking water, as well as community-level factors such as residence, region, community-level poverty, and community level media usage, had a significant association. Open defecation practice was significantly and disproportionately concentrated on the poor households [C = -0.669; 95% CI: -0.716, -0.622]. A non-random open defecation practice was observed in Ethiopia. Among the 11 regions, primary clusters were identified in only 3 regions (Afar, Somali, and Eastern Amhara)

**Conclusion:**

Open defecation practice remains a public health problem irrespective of the significant decrease seen in Ethiopia for the past 16 years. Individual and community-level factors had a significant association with this problem. Since it is a leading cause of under-five children mortality and morbidity, the Ethiopian ministry of health should plan and work on basic sanitation programs that focus on the poorest communities, rural societies, and small peripheral regions. These programs should include regional planning for sanitation, and translation of materials into local languages to prevent under-five mortality and morbidity due to diarrheal diseases caused by open defecation.

## Background

Open defecation means the disposal of human faeces in the fields, bushes, forests, open bodies of water, beaches, and other open spaces [1]. Around 892 million people worldwide still practice open defecation [2]. The number of individuals who practice open defecation decreased from 1229 million to 892 million between 2000 and 2015 globally [2]. However, in sub-Saharan African countries the problem climbed from 204 million to 220 million [3]. This might be due to high population growth and the slippage of open defecation-free (ODF)-certified communities, which refers to community members’ failure to keep fulfilling all open defecation-free criteria [4].

Studies showed that from 2005-to 2010, Ethiopia was one of the three sub-Saharan African countries next to Angola and Sao Tome and Principe that reduced open defecation practice [5,6]. However, a recent report showed that open defecation practice was increasing in the same way in sub-Saharan African countries [7]. The national open defecation rate in 2014 was 34.1% [8] and was practiced by 28.3 million in 2015 [6,9].

Poor sanitation is still a serious public health issue that has been related to several undesirable health effects [10]. The practice of open defecation (OD) aids in the transmission of microorganisms that cause diarrheal diseases [11], and children are the most vulnerable [12]. Diarrheal disease is the second major cause of death in children under the age of five, each year causing 1.7 million morbidities and 760, 000 deaths worldwide [13]. The burden is even higher in the African region [14]. In Ethiopia, diarrhea kills half a million under-five children annually [15]. Moreover, open defecation exposed hundreds of millions of girls and women to increased sexual exploitation and lack of privacy when they are menstruating [16].

OD practices are highly prevalent in rural areas of low-income countries [5] and economic inequalities between the poorest and richest had been reported as a major factor [6]. A previous study showed that per capita aid disbursement for sanitation had a strong relationship to OD reduction in low-income countries [5]. Other factors such as education status [17,18], financial status [4,18], household size [18], occupation [18], residence [6,19], and region [6] had an association with open defecation.

Interventions to improve human excreta disposal facilities have been demonstrated to be successful in preventing diarrheal diseases at their most important source by preventing human fecal contamination of water and soil [14,20]. According to the 2030 Sustainable Development Agenda, no child should die or get sick as a result of drinking contaminated water, and/or being exposed to other people’s excreta [2]. In Ethiopia, since 1995 after the government incorporated public health in the National Constitution, the sanitation program has been given special attention. Afterward, the Ministry of Health developed the National Hygiene and Sanitation Strategy and National Hygiene and On-Site Sanitation Protocol in 2005 and 2006 consecutively [21,22]. The Community-Led Total Sanitation and Hygiene (CLTSH) program was introduced in 2006 by an Irish NGO called "VITA" which was an effective strategy to reduce the practice of open defecation and achieve sanitation targets [22].

Despite these interventions, open defecation in Ethiopia is still increasing [7]. As a result, children’s death due to diarrheal disease is common [15]. However, there is no evidence at the national level regarding the contributing factors to open defecation practice in Ethiopia. It is critical to understand what factors influence the pace of improving sanitation and reducing diarrhea morbidity and mortality caused by the lack of sanitation. Therefore this study was conducted to answer the following research questions. How the 16 years’ trends (2000–2016) of open defecation practice in Ethiopia look like? Are there wealth-related inequalities to practice open defecation among households in Ethiopia? What are other contributory factors which significantly associate with open defecation practice? What is the spatial distribution of open defecation among households in Ethiopia? So answering these questions will be valuable for policymakers and program planners as preliminary evidence to plan and decide accordingly.

## Methodology

### Study design, setting, and data source

**Fig 1:**
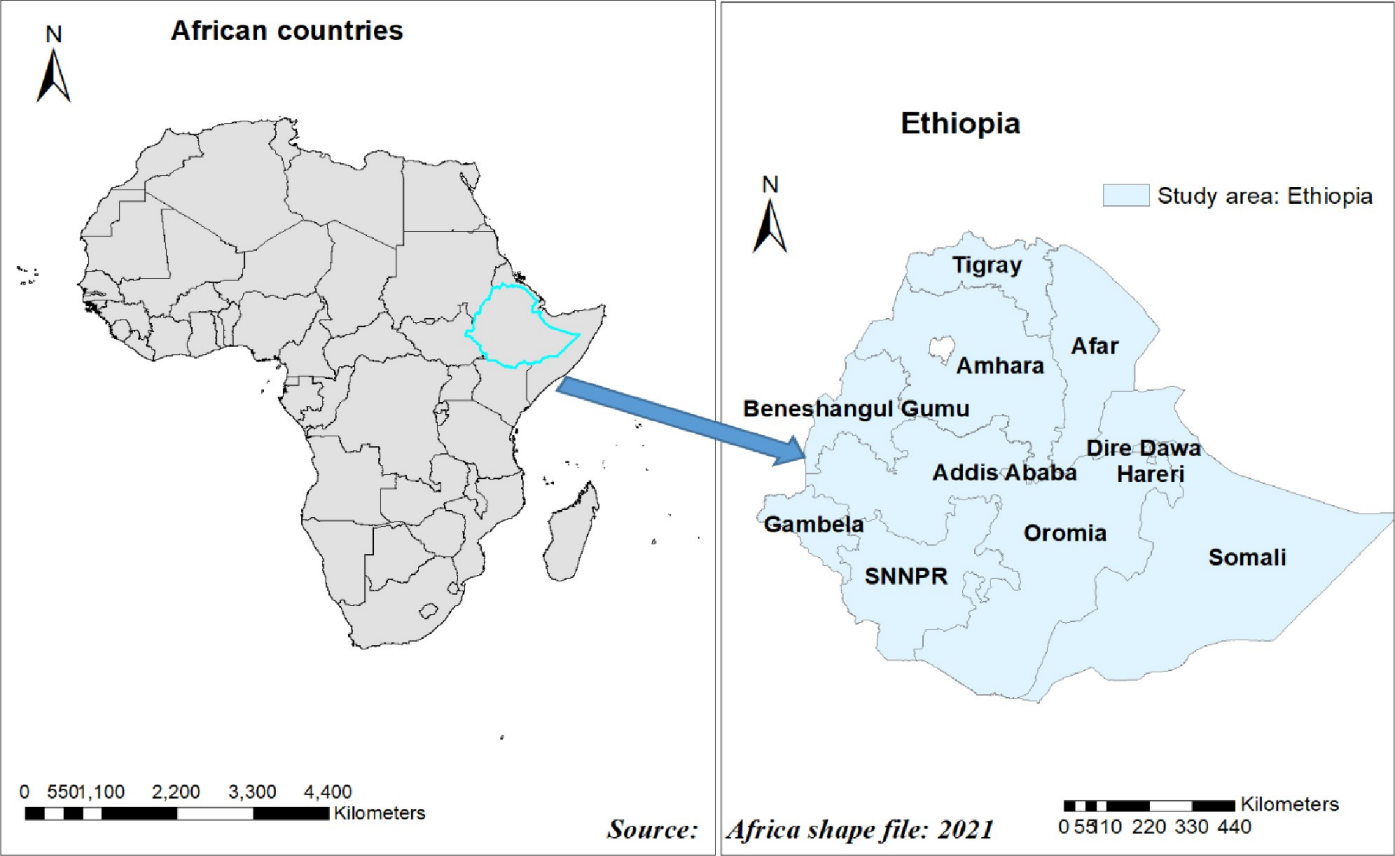
Map of Ethiopia where the surveys were undertaken within nine regions and two cities administrate plotted using ArcMap 10.7.

Population-based cross-sectional survey data from EDHS 2016 were used. Ethiopia is an East African country (3^0^–14^0^ N and 33^0^ - 48^0^E) with 1.1 million Sq. km coverage and the second-most populous nation in Africa with an estimated population of 114,963,588 in 2021 [23]. Administratively, Ethiopia is federally decentralized into nine regions (Tigray, Afar, Amhara, Oromia, Benishangul Gumuz, Somalia, South Nation Nationalities, and Peoples of Ethiopia (SNNP), Gambelia, and Harari) and two city administrations (Addis Ababa and Dire Dawa). The lowest administrative unit is the Kebele, which is subdivided into census enumeration areas (EAs). Stratified two-stage cluster sampling techniques were used in EDHS. Each region was stratified by categorizing it into urban and rural parts, and645 Enumeration Areas (EAs) were chosen in the first stage (202 in the urban area) with probability selection proportionate to EA size. On average, 25–30 households were systematically chosen in the second stage. The detail of the study design and setting is available elsewhere [24] **[Fig 1].**

### Study population

All households assessed for sanitation facilities during the survey (EDHS 2016) were the study population. A total of 16,650 households were studied in EDHS 2016. A total of 121 households with incomplete documentation of some independent variables (the time taken to get drinking water, age of household head, and educational level of household head) were dropped from further analysis As a result, we included a total weighted sample size of 16,554 households in the final analysis.

### Study variables

The outcome variables of the study were open defecation which means a lack of sanitation facility, defecating on bush or fields [25]. The independent variables of this study comprised individual-level variables including age, sex, marital status, educational attainment of household head, household family size, household wealth index, media exposure status of the households, and source of drinking water, and community-level variables including place of residence, region, community media usage, and community poverty. Media exposure was created from two variables; watching TV and listening to the radio. If a woman has exposure to at least one type of media, she was considered exposed to media [26]. Based on the development status and the need for governmental support, the 11 regions of Ethiopia are categorized into three groups; ‘three Metropolis’ (Addis Ababa, Harari, and Diredewa), large central (Tigray, Amhara, Oromia, SNNPR), and “small peripherals” (Afar, Benshangul-Gumuz, Gambelia and Somali) [26].

The level of poverty in the community was determined by the proportion of households in the poorer and poorest quintiles obtained from the wealth index results. The recoding of community aggregate factors was taken from national report percentages. For community poverty, according to the world bank (WB), 2019/ 2020 report around 24% of the population is in poverty [27]. For community mass media exposure we have used 13.8% and also for community women’s education level we used 7.7% [28]. The normal distribution of aggregated community factors was assessed by histogram and Shapiro Wilks test and then we recorded them based on the appropriate measure of central tendency.

### Operational definitions

#### Open defecation

Lack of sanitation facility, defecates on bush or field [29].

#### Unimproved sources of drinking water

A household is said to have access to an unimproved drinking water source if it had water unprotected dug well, unprotected spring, tanker truck/cart with a small tank, surface water, and others [30].

#### Data management and analysis

This study was performed based on the three EDHSs data obtained from the official DHS measure website www.measuredhs.com. The permission was received via an online request by specifying our analysis objective. The set of household record (HR) data was used to extract the outcome and the independent variables. The data clearance, descriptive, and summary statistics were conducted using STATA version 14.2 software. Before we conduct any statistical analysis, the data were weighted for the sampling probabilities using the weighting factor to restore the representativeness of the survey and to get reliable statistical estimates.

### Mixed effect analyses model building

Since the EDHS data has a hierarchical structure and households were nested within a cluster/EAs, it violated the assumption of independence of observations and equal variance across clusters. Thus mixed effect models which included both fixed and random effects were used to assess the clustering effect of open defecation usage.

The fixed effects were used to estimate the association between the likelihood of open defecation and explanatory variables at both individual and community levels. From bivariable analysis factors with a p-value ≤, 0.2 were selected as candidates for the final model. In the final multivariable analysis, the associations between dependent and independent variables were presented using adjusted odds ratios (AOR) and 95% confidence intervals (CI) with a p-value of <0.05.

Random effects were used to estimate a measure of variation using the Interclass Correlation Coefficient (ICC), Median Odds Ratio (MOR), and Proportional Change in Variance (PCV).

The ICC revealed the variation of open defecation between clusters was calculated as; $ICC=\frac{VC}{VC+3.29}*100\%$, where; VC = cluster level variance

The MOR is defined as the median value of the odds ratio between the area at the lowest risk and at the highest risk when randomly picking out two clusters.

MOR = exp.[√(2 × VC) × 0.6745], or ${{MOR=e}^{0.95}}^{\sqrt{VC}}$ where; VC is the cluster level variance.

The PCV showed the variation in open defecation among households explained by both individual and community-level factors. $PCV=\frac{Vnull-VC}{V\;null}*100\%$ where; Vnull = variance of the initial model, and VC = cluster level variance of the next model [31–33].

Generally, in mixed-effect analysis, four models were fitted. The first was the null model, which contained only the outcome variables to check the variability of open defecation in the cluster. The second and the third multilevel models contain household-level variables and community-level variables respectively, while in the fourth model both household and community level variables simultaneously were fitted with the open defecation. Model comparison was done using the deviance test and the model with the lowest deviance was selected as the best-fitted model [31–33].

### Concentration index and graph analyses

The concentration index and graph approach were used to examine socioeconomic inequalities in health outcomes [34,35]. The concentration curve was applied to identify whether there was socioeconomic inequality in some health variables or it was more pronounced at one point. It displays the share of health accounted by cumulative proportions of individuals in the population ranked from the poorest to the richest [35,36].

The concentration curve would be a 45^0^ line indicating the absence of inequity while, the concentration curve lying above and below the equality line (45^0^) indicated that open defecation practice is disproportionately concentrated between poor and rich, respectively [37]. The greater the degree of inequity, the more the concentration curve diverged from the diagonal line [35].

Twice the area between the concentration curve and the diagonal line is the concentration index [36,38]. It ranges from −1 to + 1 and the sign indicates the direction of the relationship between the health variable (open defecation) and the distribution of living standards (wealth status) [35,39].

### Spatial analyses

The weighted proportion of open defecation data was exported to ArcGIS 10.7 software and spatial distribution, spatial autocorrelation, incremental autocorrelation, spatial interpolation, and detection of hot spot areas were analyzed. Spatial scan statistics were employed using Kuldorff’s SaTScan version 9.6 software [40].

#### Spatial autocorrelation and interpolation analyses

Spatial autocorrelation (Global Moran’s I) statistic measure was used to assess whether open defecation among households in Ethiopia was dispersed, clustered, or randomly distributed [41].

The spatial interpolation technique was used to predict open defecation in unsampled households based on sampled clusters. The geostatistical ordinary kriging spatial interpolation technique was used for the prediction of unsampled clusters using ArcGIS 10.7 software.

#### Hot spot analysis and spatial scan statistics

Hotspot analysis showed that, the features with either hot spot or cold spot areas for open defecation. The hot spot areas indicated that there was a high proportion of open defecation among households and the cold spot ones indicated that there was a low proportion. The proportion of open defecation among households in each cluster was taken as an input for hotspot analysis.

Bernoulli-based model spatial scan statistics were employed to determine the geographical locations of statistically significant clusters for open defecation among households using Kuldorff’s SaTScan version 9.6 software [40]. The scanning window that moved across the study area in which households had open defecation were taken as cases and those households which had toilet defecation (improved or unimproved) were taken as controls to fit the Bernoulli model. The circle with the highest LLR test statistic was defined as the most likely (primary) cluster. For each identified cluster, the log-likelihood ratio (LLR) test statistic with its p-value, the relative risk (RR), the location radius, population, and cases was reported.

### Ethical clearance

The data sets were downloaded with permission from the “Measure DHS program” by requesting them after explaining the purpose of the study. For anonymity purposes, the data set had no individual names or household addresses.

## Result

### Socio-demographic characteristics of the study population

A total weighted 16,554 households were included in this study. Of these, three fourth 12,351 (74.61%) of the household heads were males. Most (79.71%), of the study participants, were living in rural areas and more than half 9,047(54.65%) of the head of household had no formal education.

Among the total weighted (16,554) households, one-third (32.23%) used open defecation while the rest 11,219 (67.77%) used toilet facilities. In small periphery regions, more than half (51.1%) of the households used open defecation whereas only a small number (4.33%) of households used open defecation in large metropolitan cities. Three-fifths of the study households (61.51%) have improved sources of drinking water **[Table 1].**

**Table 1:** Socio-demographic characteristics of the study population with open defecation usage in Ethiopia, 2016 EDHS.

Variables	Categories	Open defecation		Total weighted frequency (%)
		Yes (%) n = 5,335 (32.23)	No (%) n = 11,219 (67.77)	
**Age of household head (years)**	10–19	51 (26.06)	145(73.94)	196 (1.18)
	20–29	893 (31.67)	1,925 (68.33)	2,818(17.02)
	31–39	1,362 (32.12)	2,879 (67.88)	4,240 (25.62)
	40–49	1,064 (33.27)	2,133 (66.73)	3197 (19.31)
	50–59	732 (32.73)	1,503 (67,27)	2, 225 (13.50)
	≥ 60	1,234 (31.90)	2,634 (68.10)	3,868 (23.37)
**Sex of household head**	Male	3,919 (31.73)	8,432 (68.27)	12,351 (74.61)
	Female	1,416 (33.68)	2,788 (66.32)	4,203 (25.39)
**Educational attainment** **of household head**	No education	3,772 (41.69)	5,275 (58.31)	9,047 (54.65)
	Primary education	1,332 (26.55)	3,687 (73.45)	5,019 (30.32)
	Secondary & above	230 (9.26)	2,257 (90.74)	2,488 (15.03)
**Marital status of head of household**	Married	4,109 (32.54)	8,520 (67.46)	12,629 (76.29)
	Not married	1,225 (31.22)	2,699 (68.78)	3,925 (23.71)
**House hold family size**	1–3	1,738 (31.40)	3,797 (68.60)	5,535 (33.43)
	4–6	2,390 (31.85)	5,114 (68.15)	7,504 (45.33)
	7 & above	1,207 (34.32)	2,309 (65.68)	3,516 (21.24)
**Media exposure**	No	4,415 (40.85)	6,393 (59.15)	10,808 (65.29)
	Yes	920 (16.01)	4,827 (83.99)	5,747 (34.71)
**Wealth index**	Poor	3,993 (62.79)	2,366 (37.21)	6,360 (38.42)
	Middle	745 (23.97)	2,363 (76.03)	3,109 (18.78)
	Rich	596 (8.42)	6,489 (91.58)	7,086 (42.80)
**Source of drinking water**	Improved	2,536 (24.90)	7,647 (75.10)	10,183 (61.51)
	Un improved	2,799 (43.94)	3,572 (56.06)	6,371 (38.49)
**Community-level variables**				
**Residence**	Urban	232 (6.91)	3,127 (93.09)	3,359 (20.29)
	Rural	5,103 (38.67)	8,092 (61.33)	13,195 (79.71)
**Region**	Metropolis	38 (4.33)	845 (95.67)	3,760 (22.75)
	Large centrals	4,853 (32.79)	9,949 (67.21)	7,482 (45.27)
	Small periphery	444 (51.1)	424 (48.9)	5,287 (31.99)
**Community media usage**	low	4,034 (46.150	4,706 (53.85)	8,302 (50.23)
	high	1,301 (16.65)	6,513 (83.35)	8,227 (49.77)
**Community poverty level**	low	1,493 (17.35)	7,110 (82.65)	8,263 (49.99)
	high	3,842 (48.32)	4,109 (51.68)	8,266 (50.01)

### The trend of open defecation practice in Ethiopia

**Fig 2:**
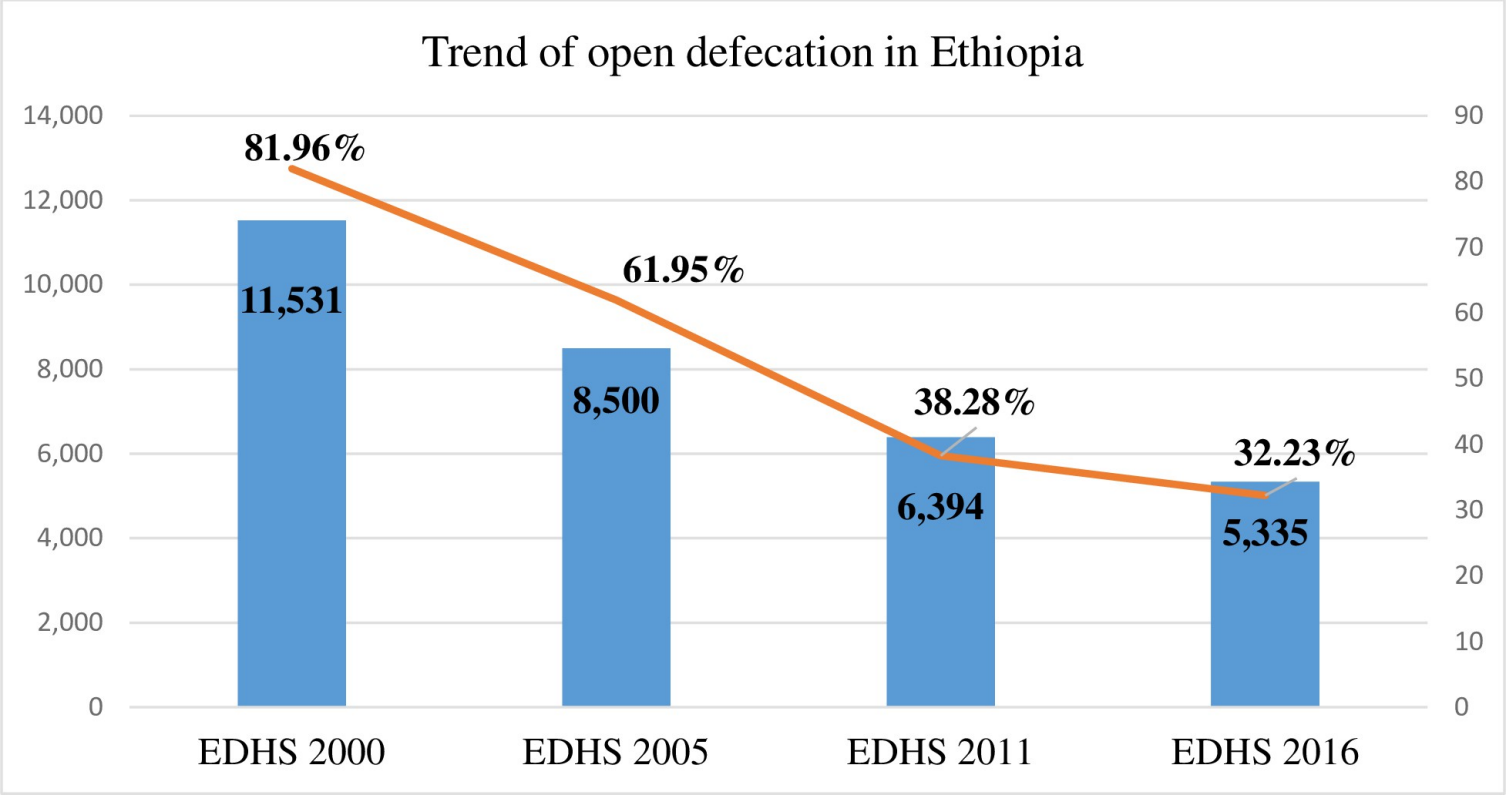
The trend of open defecation practice in Ethiopia.

The overall trend of open defecation practice in Ethiopia had significantly decreased from 81.96% (95% CI: 81.08, 82.84) in 2000 EDHS, to 32.23% (95% CI: 31.16, 33.31) in 2016 EDHS **[Fig 2]**. In 2005 EDHS and 2011 EDHS it was 61.95% (95% CI: 60.89, 63.00) and 38.28% (95% CI: 37.18, 39.39) respectively. The difference in confidence interval did not overlap in either of the phases (2000–2005, 2005–2011, 2011–2016) which indicated that the change in the proportion of open defecation was significant in each phase.

### Mixed effect model parameter results

#### Random effect and model comparison

The ICC in the null model of Table 3 showed that about 62% of the variations of open defecation practices among study households were attributed to the difference at the cluster level, the rest 38% were attributed to individual households factors.

The MOR value was 16.93, in the null model, which also revealed the median odds of using open defecation between the lowest and the highest open defecate clusters.

Furthermore, the PCV value in the final model (30%) indicated the variation in the open defecation usage among study households, and this was explained by both the individual and community level factors simultaneously. Model comparison/fitness was done using the deviance test, then the third model has the lowest deviance (11772) and was taken as the best-fitted model **[Table 2].**

**Table 2:** Mixed effect analysis of factors associated with open defecation among households in Ethiopia, data from 2016 EDHS.

Variables	Categories	Null model	Model 1	Model 2	Model 3
			AOR [95% CI]	AOR [95% CI]	AOR [95% CI]
Age of household head (years)	10–30		1.00	**------------**	1.00
	31–40		1.01[0.88, 1.19]	**------------**	1.04 [0.89, 1.23]
	41–59		**0.78[0.66, 0.91]** **	**------------**	**0.79[0.68, 0.93]** **
	≥ 60		**0.68[0.58, 0.81]** ***	**------------**	**0.69[0.59,0.82]** ***
Sex of household head	Male		1.00	**------------**	1.00
	Female		1.107[0.95, 1.29]	**------------**	1.12[0.96,1.31]
Educational attainment of household head	No education		1.00	**------------**	1.00
	Primary education		**0.82[0.72, 0.93]** **	**------------**	**0.84[0.74, 0.95]** **
	Secondary & above		**0.58[0.47, 0.72]** ***	**------------**	**0.63 [0.51,0.78]** ***
Marital status of head of household	Married		1.00	**------------**	1.00
	Not married		**1.19[1.00, 1.40]** *	**------------**	**1.22[1.03,1.45]** *
Household family size	1–3		1.00	**------------**	1.00
	4–6		**0.80[0.71, 0.91]** **	**------------**	**0.79[0.69,0.89]** ***
	7 & above		**0.73[0.62, 0.86]** ***	**------------**	**0.71[0.59, 0.83]** ***
Media exposure	No		1.00	**------------**	1.00
	Yes		**0.28[0. 25, 0.29]** **	**------------**	**0.44 [0.36, 0.54]** **
Wealth index	Poor		1.00	**------------**	1.00
	Middle		**0.14[0.12, 0.16]** ***	**------------**	**0.14[0.12, 0.16**]***
	Rich		**0.04[0.03, 0.04]** ***	**------------**	**0.05[0.04, 0.50**]***
Source of drinking water	Improved		1.00	**------------**	1.00
	Un improved		**1.18[1.04, 1.33]** *	**------------**	**1.14[1.00, 1.29]** *
**Community-level variables**					
Residence	Urban		**------------**	1.00	1.00
	Rural		**------------**	**4.59[2.64, 7.99]** ***	**1.82[1.01, 3.26]** *
Region	Metropolis		**------------**	1.00	1.00
	Large central		**------------**	**2.07[1.09, 3.86]** *	**1.99[1.03, 3.85]** *
	Small periphery		**------------**	**3.09[1.58, 6.09]** **	**2.08[1.02, 4.25]** *
Community media usage	Low		**------------**	1.00	1.00
	High		**------------**	**0.38[0.26, 057]** ***	**0.51[0.33, 0.77]** **
Community poverty usage	Low		**------------**	1.00	1.00
	High		**------------**	**5.13[3.39,7.75]** ***	**1.59[1.03, 2.45**]*
**Random effect**					
	**ICC**	0.622	0.461	0.457	0.444
	**MOR**	16.93	1.96	1.51	1.49
	**PCV**	Reff	0.083	0.292	0.302
**Model comparison**					
	**Deviance**	13185	11846	14082	11772
	**Mean VIF**	---	1.60	1.82	1.77

* = P-value < 0.05

** = Pvalue < 0.01

*** = Pvalue < 0.001.

ICC = Inter cluster corrolation cofficent, MOR = Median odds ratio, PCV = proportional change in variance. AOR = adjusted odds ratio; CI = confidence intervalm.

**Table 3:** Significant spatial clusters of open defecation practice among households in Ethiopia, EDHS 2016.

Clusters	Enumeration areas (clusters) detected	Coordinate/radius	Population	Cases	RR	LLR	P-value
1* [174]	421, 384, 511, 605, 130, 172, 220, 550, 237, 94, 160, 99, 79, 298, 623, 585, 235, 424, 430, 127, 196, 128, 538, 362, 143, 449, 392, 129, 263, 226, 341, 136, 442, 575, 355, 488, 604, 134, 117, 192, 300, 579, 351, 542, 97, 481, 599, 404, 598, 249, 461, 103, 544, 45, 156, 413, 344, 84, 455, 636, 200, 332, 551, 66, 81, 89, 590, 400, 425, 241, 597, 479, 348, 401, 478, 389, 584, 340, 188, 628, 427, 189, 181, 591, 80, 199, 571, 496, 191, 98, 255, 528, 410, 322, 152, 78, 583, 611, 205, 258, 312, 327, 178, 499, 345, 254, 368, 627, 570, 268, 354, 545, 334, 18, 616, 640, 638, 55, 163, 132, 612, 512, 440, 632, 38, 617, 596, 547, 366, 296, 4, 158, 456, 253, 276, 176, 504, 460, 75, 620, 292, 120, 169, 73, 10, 24, 279, 403, 167, 283, 206, 267, 431, 310, 382, 429, 37, 516, 637, 102, 135, 482, 510, 295, 229, 336, 572, 361, 350, 375, 39, 531, 474, 484	13.342722N, 39.759716E/ 402.87 km	4475	2523	2.18	645.6	<0.0001
2 [23]	266, 618, 309, 435, 536, 370, 507, 592, 104, 260, 233, 69, 426, 603, 346, 315, 567, 343, 13, 105, 106, 417, 284	8.389747N, 33.258557E/ 138.81 km	587	525	2.77	403.8	<0.0001
3 [53]	490, 543, 92, 492, 171, 198, 146, 95, 85, 358, 164, 138, 497, 521, 588, 458, 553, 278, 77, 629, 214, 318, 251, 573, 187, 239, 116, 22, 33, 568, 277, 527, 269, 556, 378, 630, 64, 439, 57, 480, 8, 210, 186, 454, 436, 566, 212, 501, 513, 68, 622, 1, 580	6.745502N, 44.259011 E / 366.72 km	1249	746	1.85	181.6	<0.0001

* = primary clusters.

#### Mixed effect analysis of factors associated with open defecation

All variables which had a p-value <0.20 in the bivariable analysis were eligible for multivariable analysis. Based on the final model result, individual-level variables such as the age of household head, educational attainment of household head, marital status of head of household, household family size, media exposure, wealth index, source of drinking water had a significant association with open defecation practice. Among the community-level factors residence, region, community-level poverty, and community-level media usage were found to be significantly associated with open defecation.

As the age of houshold head increased to 41–59 and ≥60, the odds of OD usage decreased by 21% and 31% [AOR = 0.79; 95%CI; 0.68, 0.93] and [AOR = 0.69; 95%CI; 0.59, 0.82] respectively.

The odds of using OD decreased by 16% and 37% as the educational status of the head of household increased to primary and above primary educational status [AOR = 0.84;95%CI;0.74, 0.95] and [AOR = 0.63;95%CI; 0.51,0.78] respectively.

As the family members of the household increased to 4–6 and above six the odds of using OD decreased by 21% and 29% [AOR = 0.79;95%CI; 0.69,0.89] and [AOR = 0.71;95%CI; 0.59, 0.83] respectively.

Households who had media exposure and high community media usage were 56% and 49% less likely to use OD as compared to none exposed and low community media usage [AOR = 0.44;95%CI; 0.36, 0.54] and [AOR = 0.51;95%CI; 0.33, 0.77]. People who lived in rural households were 1.82 times more likely to use OD as compared to urban residents [AOR = 1.82; 95%CI; 1.01, 3.26].

Being middle and high wealth status were 86% and 95% less likely to had OD as compared to poor households [AOR = 0.14;95%CI; 0.12,0.16] and [AOR = 0.05;95%CI; 0.04, 0.50] respectively. Whereas those in high community poverty were 1.59 times more likely to use OD as compared to low community poverty [AOR = 1.59; 95%CI; 1.03, 2.45].

Households who have unimproved drinking water were 1.14 times more likely to use OD than their counterparts [AOR = 1.14; 95%CI; 1.00,1.29]. Living in the small periphery and large central regions were nearly two times more likely to use OD as compared to metro pollutant cities [AOR = 2.08;95%CI;1.02,4.25] and [AOR = 1.99;95%CI;1.03,3.85] respectively **[Table 2].**

### Wealth related inequality of open defecation

**Fig 3:**
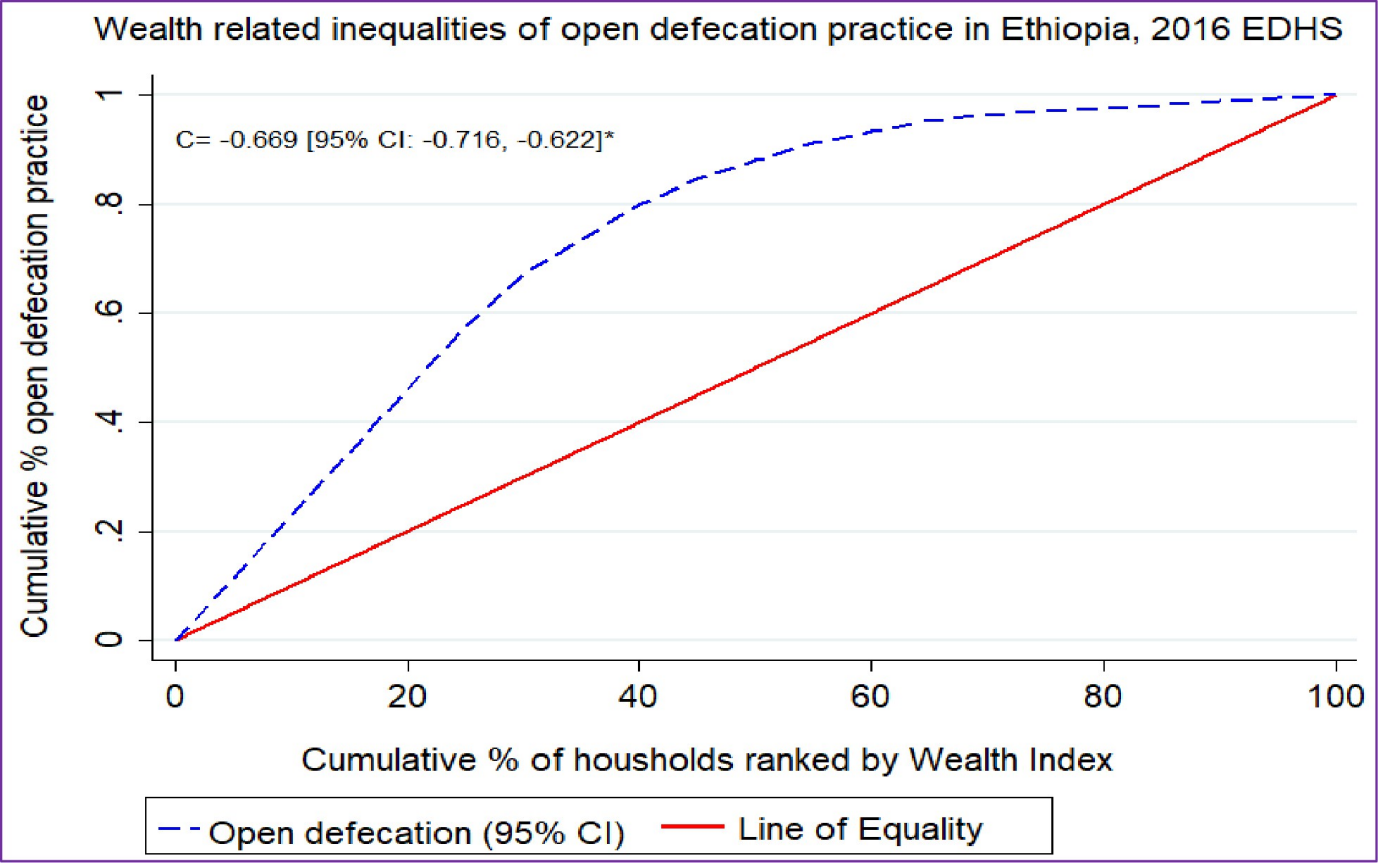
Wealth-related inequality of open defecation in Ethiopia, EDHS 2016.

In this study, the wag staff normalized concentration index (C) and curve were done for the last EDHS (EDHS 2016) to assess the wealth-related inequality of open defecation among households in Ethiopia. The result showed that open defecation was significantly and disproportionately concentrated in the poor households (pro-poor distribution) with [C = -0.669; 95% CI: -0.716, -0.622]. The graph in **Fig 3** also showed that the distribution line of open defecation was above the line of equality. This showed that open defecation among households in Ethiopia was disproportionately concentrated in the poor household (pro-poor distribution**) [Fig 3].**

### Spatial and incremental autocorrelation analysis of open defecation among households in Ethiopia: based on 2016 EDHS

**Fig 4:**
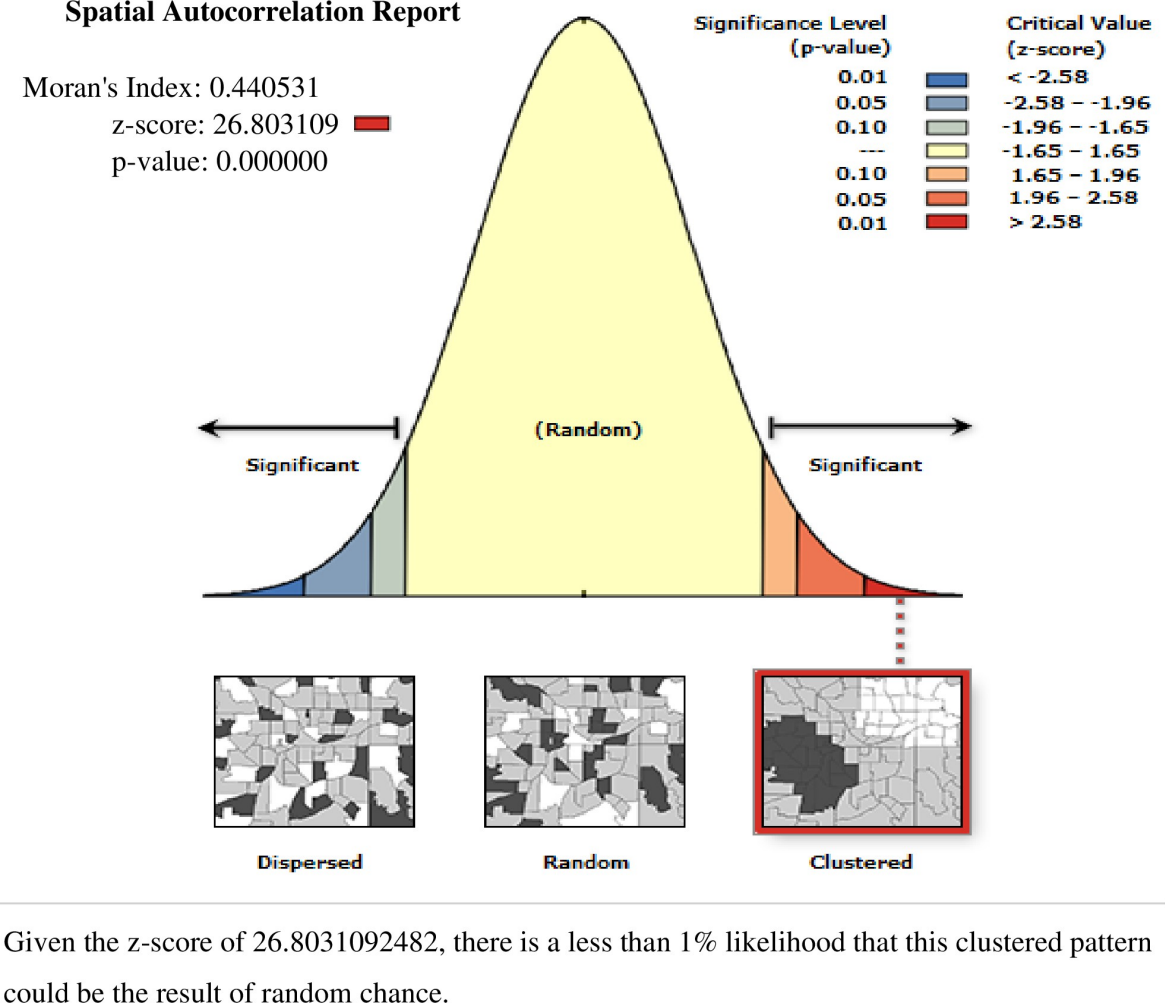
Spatial autocorrelation of open defecation usage among households in Ethiopia, EDHS 2016 plotted using ArcMap 10.7.

Spatial distribution of open defecation usage among households in Ethiopia based on 2016 EDHS showed a significant spatial variation across the country over regions, which was found to be non-random with Global Moran’s I value of 0.44 with (p< 0.0001) **[Fig 4].**

The incremental autocorrelation result showed that statistically significant z-scores indicated at one peak distance at 151.378Km; 32.13(distances; Z-score) for open defecation use, where spatial processes promoting clustering were most pronounced by 10 distance bands.

### Spatial distribution and interpolation of open defecation in Ethiopia

**Fig 5:**
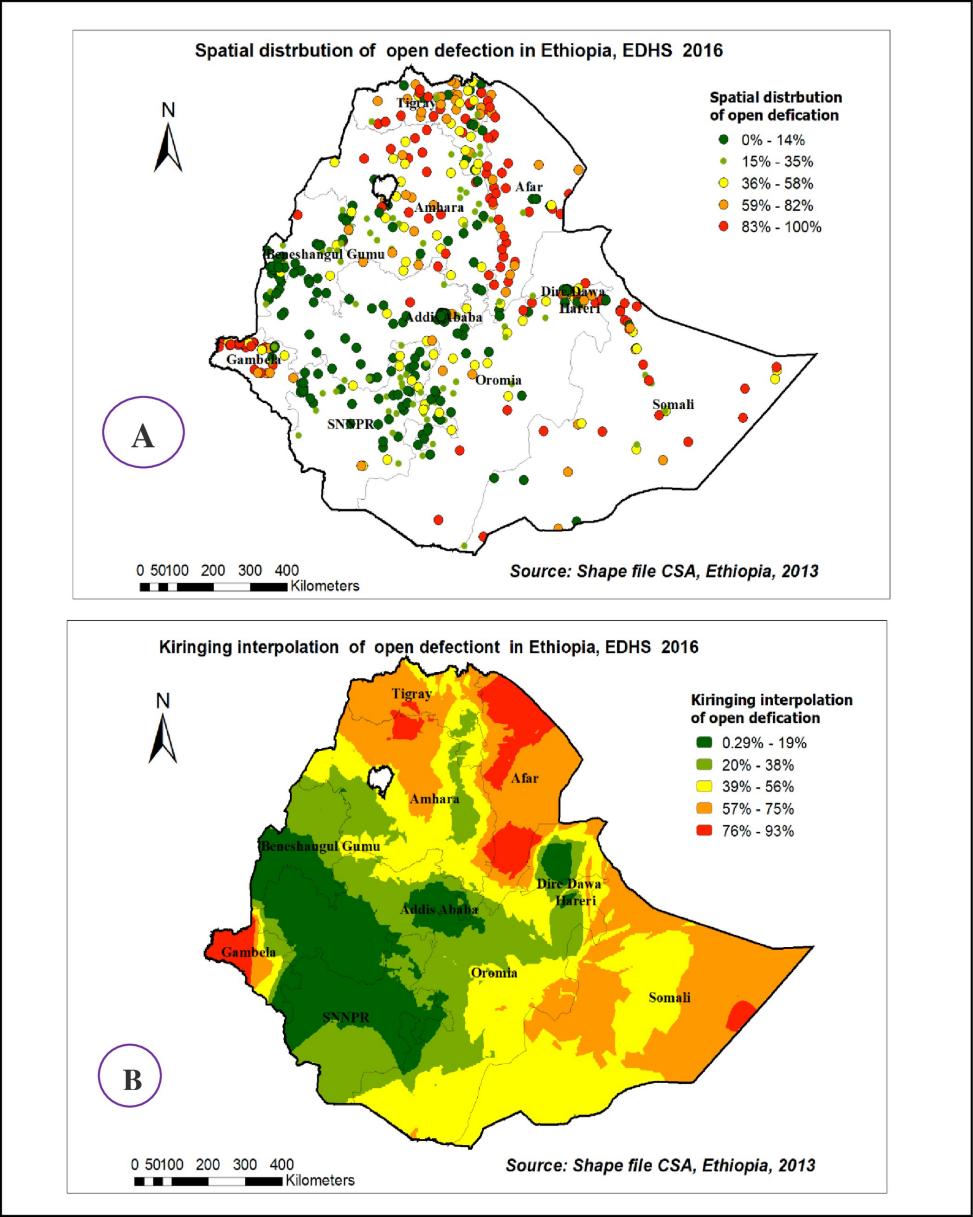
Spatial distribution (A) and interpolation(B) of open defecation in Ethiopia EDHS 2016 plotted using ArcMap 10.7.

As shown in the following figures, the red dots indicated the more intense clustering of the proportion of open defecation among households in Ethiopia, whereas the green dots showed a lower proportion of the problem **[Fig 5(A)].** Fig 3B, showed Kriging interpolation methods of predicting open defecation among households in Ethiopia over the area which was increased from green which indicates low- risk to red-colored which indicates high-risk areas. The prevalence of high-risk areas predicted for open defecation was extremely high (ranging from 76% to 92%) in Tigray, Afar, Northern Amhara, Gambelia, and Somalia regions. Whereas the lower predicted open defecation was seen in Addis Ababa, Dire Dawa, Harari, Benishangul Gumuz, and SNNP (south nation nationalities and peoples of Ethiopia) regions **[Fig 5(B)].**

### Hot spot analysis (Getis-Ord Gi* statistic) of open defecation practice in Ethiopia

**Fig 6:**
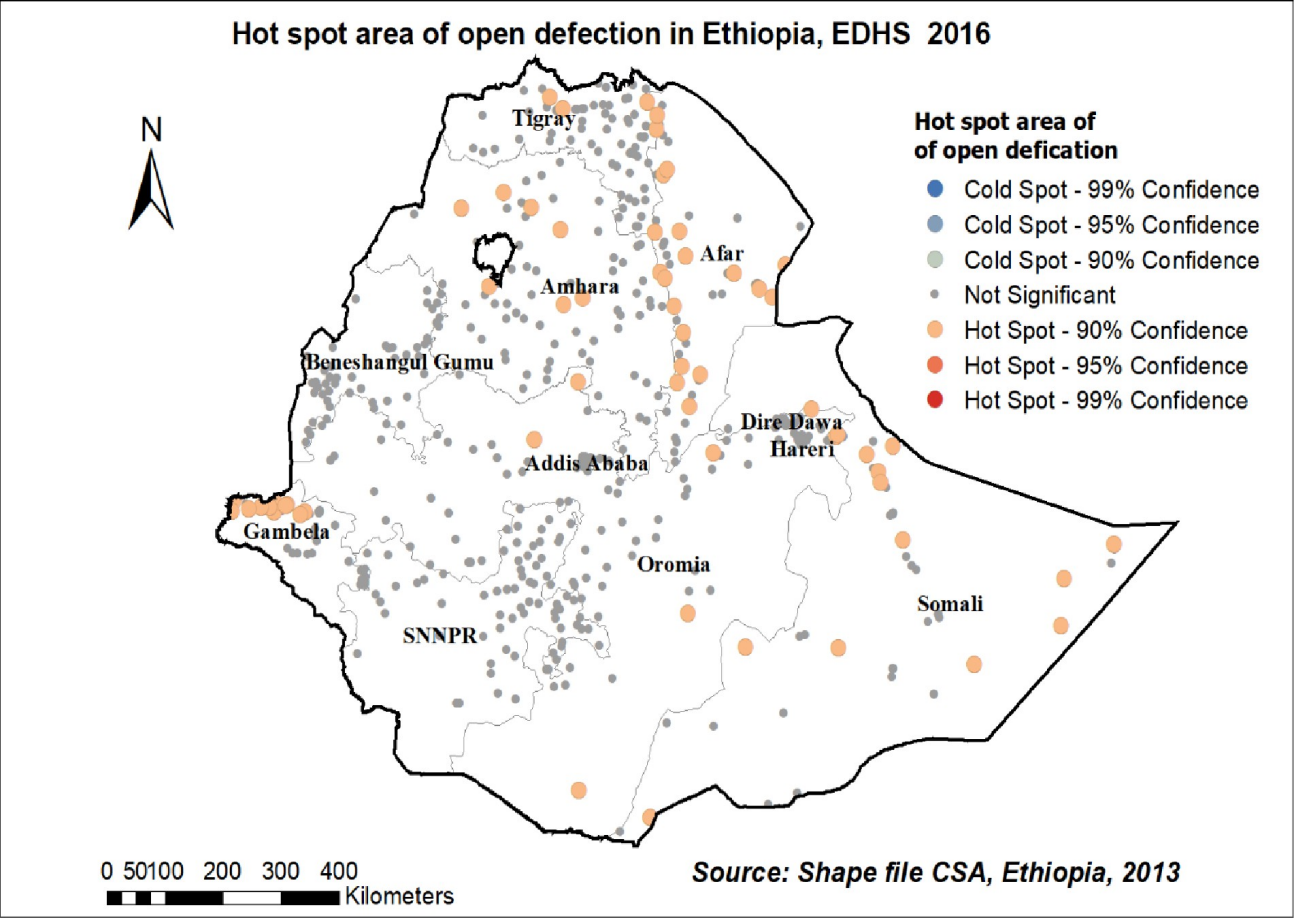
Hot spot area of open defecation in Ethiopia EDHS 2016 plotted using ArcMap 10.7.

The spatial distribution of open defecation practice in 2016 EDHS showed that hot spot areas were detected in western Afar, Eastern Amhara Gambelia, and Somalia regions, while cold spot areas were detected in Addis Ababa and Diredewa **[Fig 6].**

### Spatial SaTScan Statistics Analysis of open defecation practice in Ethiopia

**Fig 7:**
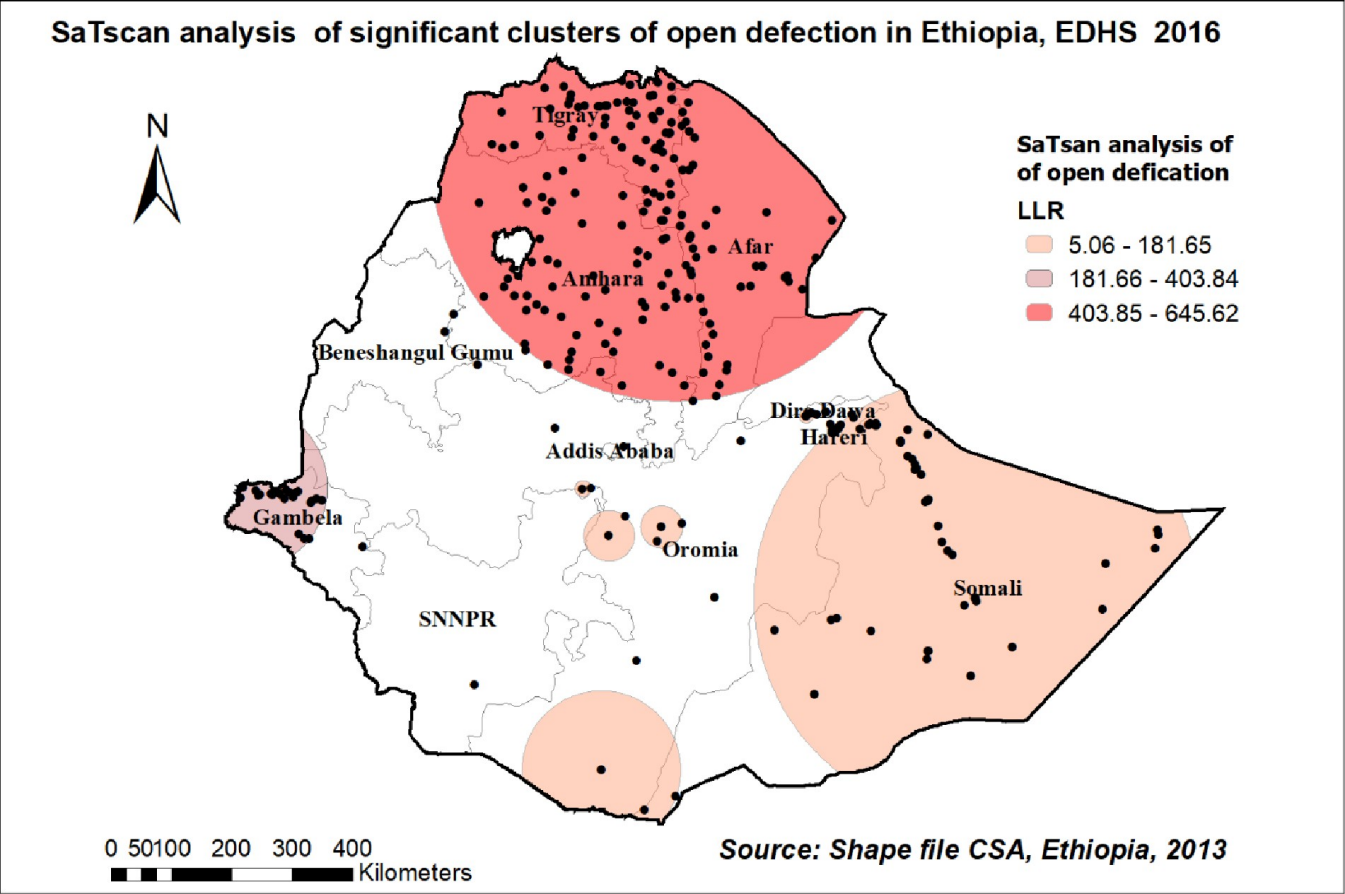
Significant clusters of open defecation spatial window in Ethiopia, EDHS 2016 plotted using ArcMap 10.7.

There were primary and secondary clusters of open defecation practices among households in Ethiopia. Among the total of 250 clusters, 174 were primary clusters. These were located in the entire Afar, Tigray, and most of Amhara regions centered at 13.342722N, 39.759716E with a 402.87 km radius. Households found in the SaTScan window were two times more likely to use open defecation (RR = 2.18, P-value<0.0001) **[Table 3, Fig 7].**

## Discussions

This study was conducted to assess the trend, spatial distribution, and determinants of open defecation, among households in Ethiopia. This study showed that open defecation practice in Ethiopia had been significantly decreased from 81.96% (95% CI: 81.08, 82.84) in 2000 EDHS, to 32.23% (95% CI: 31.16, 33.31) in 2016 EDHS. This is in line with the WHO report that Ethiopia was one of the three sub-Saharan African countries which decreased open defecation by 10% or more [5,6]. The WHO and UNICEF Joint Monitoring Program data also showed that Ethiopia tremendously decreased open defecation between the years 2000 and 2015 [2]. This might be due to the involvement of different community initiative programs such as the Community-Led Total Sanitation and Hygiene program an Irish NGO, which had a better approach toward the reduction of open defecation practice and the achievement of the desired sanitation program [4,22].

In this study as the age of the household head increased, OD practice decreased. This is in agreement with a study in rural North India [42]. The reason might reflect that as age increases, disability or incontinence might occur, which makes open defecation difficult or impractical [42]. Moreover, older people on average are unable to move more freely outside their homes to practice open defecation.

In this study, open defecation practice decreased as the educational status of the household head increased. This is supported by a study in rural Tanzania [43], Ghana [18], Nigeria [44], and a systematic review and meta-analysis in Ethiopia [17]. A study in Ghana showed that households with an educated head were 18.5% less likely to practice open defecation [18]. This could be because educated household heads might understand better the effects of open defecation and the relevance of having sanitation facilities. A higher level of education increases the income earning capacity of a household and thereby expanding their capacity to construct a toilet facility [45].

In our study, contrary to other studies, as the number of family members in the household increased the odds of using OD decreased. But a study in Ghana showed that households with large sizes were 40% more likely to defecate in the open than those with smaller sizes [18]. The difference could be explained by household members’ charactersticses such as education status, age, and wealth status.

In this study, households who had media exposure and high community media usage were less likely to use OD. It is supported by a study in India [46], and in Nigeria showed that exposure to mass media, social media, and community-based media helped to prevent OD practice [47]. This might be that exposure to mass media increases awareness about the negative impacts of open defecation practice [44,46].

Households who had unimproved drinking water were more likely to use open defecation. Supported by a study in Dangilla Ethiopia, where water access had an association with OD practice [8]. This could be explained by the fact that households had water shortages and unimproved water sources could not keep their hygiene due to lack of water, especially for toilet use.

In this study, having middle and high wealth status in the household was associated with less OD practice as compared to poor households whereas, households who lived in high community poverty were more likely to use OD. The concentration index and graph also showed that open defecation was significantly and disproportionately concentrated in poor households. This was in line with studies in Ethiopia [4], Nigeria [44], and Gahanna [18]. The majority of OD practices were found in rural areas of low-income countries [5], and there were economic inequalities in OD practices between the poorest and richest [6]. Those countries with high levels of poverty had widely practiced open defecation, and there was a large disparity between the rich and poor [18,48]. A study showed that per capita aid disbursement for sanitation had a strong relationship to OD reduction in low-income countries [5].

In this study, people who live in rural households were more likely to use OD as compared to urban. This is in line with a study done in Nigeria [44], India [49], Nepal, and [6], WHO reports [6]. This might be due to an unequal distribution of power and limited access to infrastructure, information, and income which leads to poor practices of open defecation and limited sanitation in rural residences [44].

The mixed-effect analysis of this study showed that living in the small periphery and large central region were nearly two times more likely to use OD as compared to metropolitan cities. The spatial distribution and spatial clustering windows also showed that significant clustering had been detected in Afar, Gambella, and Somali regions which were under small peripheral groups. This was in line with a study in Nigeria [44], a previous study in Ethiopia in Afar, Somali, and Gambella where open defecation was predominant [50]. This is because these regions are the highest proportion of pastoralist communities and comparatively low commitment to planning for open defecation-free outcomes. Since they are mobile societies, there is tendency for resistance to building and using latrines. Hence, creation of an open defecation environment is envisaged [9]. A high prevalence of drought and water-related conflicts are also likely important factors for the poor hygiene and sanitation coverage in the region [50].

The main strength of this study was the use of the weighted nationally representative data with a large sample which made it a good representative at national and regional levels. Therefore, it can be generalized to all households during the study period in Ethiopia. Moreover, the use of a mixed effect model that took into account the nested nature of the EDHS data and the variability within the community to get a reliable estimate and standard errors. But it is not free of limitations mainly resulting from the use of secondary data. As some important confounders like the amount of water they get and behavioral factors are missed. Moreover, recall biases and social desirability biases might be expected.

## Conclusion

Open defecation practice remains a public health problem irrespective of the significant decrease seen in Ethiopia for the past 16 years. Individual-level factors such as; age, educational attainment, marital status, media exposure, wealth status, and source of drinking water, as well community-level factors such as residence, region, community-level poverty, and community level media usage had a significant association with open defecation. There is a significantly disproportional pro-poor distribution of open defecation practice in Ethiopia which means that its distribution favors poor households. Non-random open defecation was seen in Ethiopia with primary clusters identified in only Afar, Somali, and Eastern Amhara regions from the total mentioned 11 regions. The Ministry of the health of Ethiopia should plan and work on a basic sanitation and hygiene program that will focus on the poorest communities, rural societies, and small peripheral regions. Media exposure and education should be strengthened. The need for the policymakers and program planners to use this evidence in planning and decision is strongly advocated.

## Abbreviations

AOR: Adjusted Odds Ratio
CI: Confidence Interval
CSA: Central Statistical Agency
EDHS: Ethiopian Demographic and Health Survey
HR: Household Record
MOH: Ministry Of Health
OD: Open Defecation
SDG: Sustainable Development Goal
